# Effect of non-invasive transcutaneous auricular vagus nerve stimulation on cerebral motor excitability—Study protocol for a randomized, sham controlled trial

**DOI:** 10.3389/fneur.2023.1341898

**Published:** 2024-01-12

**Authors:** Thorsten Herr, Paula Kleger, Sebastian Strauss, Christoph Szeska, Nura Khalil, Bashar W. Badran, Mathias Weymar, Matthias Grothe

**Affiliations:** ^1^Department of Neurology, University Medicine Greifswald, Greifswald, Germany; ^2^Department of Biological Psychology and Affective Science, Faculty of Human Sciences, University of Potsdam, Potsdam, Germany; ^3^Department of Psychiatry and Behavioral Sciences, Neuro-X Lab, Medical University of South Carolina, Charleston, SC, United States; ^4^Faculty of Health Sciences Brandenburg, University of Potsdam, Potsdam, Germany

**Keywords:** taVNS, motor cortex excitability, TMS, neurophysiology, vagal nerve

## Abstract

Transcutaneous auricular vagus nerve stimulation (taVNS) is becoming increasingly established in the treatment of various neurological and psychiatric diseases. However, only a few studies have focused on the overall influence of taVNS on cortical excitability in general. The planned study will investigate the effect of taVNS on the excitability of the motor cortex in young healthy subjects. The aim of the study is to gain better understand of the physiological mechanism of taVNS to contribute to new fields of application of taVNS in new areas such as the treatment of stroke or multiple sclerosis. This protocol describes a single-center, prospective, double blind, sham-controlled trial that evaluates the effect of taVNS on motor cortex excitability with a planned sample size of 30 participants. The effect of taVNS is investigated by neuronavigation and electromyography (EMG) coupled transcranial magnetic stimulation (TMS) applied before and after taVNS stimulation. The following parameters are assessed: resting motor threshold (RMT), active motor threshold (AMT), recruitment curve (RC), short intracortical inhibition (SICI), intracortical facilitation (ICF). All parameters will be assessed for taVNS on the basis of perception threshold and tolerance threshold. All investigations performed in the study were reviewed and approved by the local ethics committee of the University Medical Center Greifswald (study reference number: BB048/22).

**Clinical trial registration:**

www.drks.de, number: DRKS00029937.

## Introduction

Transcutaneous auricular vagus nerve stimulation (taVNS) is a rapidly developing technique for non-invasive neuromodulation that was repeatedly used to both improve health and mitigate health burden (1). While the stimulators were initially implanted invasively, they are increasingly replaced by non-invasive stimulators which first studies used to treat neurological and psychiatric disorders with (2, 3). The non-invasive, transcutaneous stimulation of the auricular branch of the vagus nerve activates the nucleus tractus solitarii (4–6). This brainstem activation leads to a broad distribution of afferent information within several cortical and subcortical areas, including the serotonergic raphe nuclei, the cholinergic nucleus basalis Meynert the pedunculopontine nucleus, and the noradrenergic locus coeruleus (7–9). Accordingly, there is evidence that taVNS leads to modulation of the neurotransmitter system including the serotonergic, cholinergic and noradrenergic system, but also GABAergic systems (10–12). This widespread activation can be demonstrated with functional magnetic resonance imaging (fMRI) (10–13). After taVNS application not only subcortical areas such as the locus coeruleus and the nucleus tractus solitarii but also cortical areas like the postcentral gyrus, cerebellum, prefrontal cortex, insula, or anterior cingulate are activated (10, 13). This cortical modulation is considered a possible explanation of the reduction in seizure frequency in epilepsy patients treated with taVNS (14, 15). The beneficial effect of VNS on post-stroke recovery also suggests a modulation on the motor system, but very few studies have focused on this (16).

The cortical excitability of the primary motor system can be investigated non-invasively with transcranial magnetic stimulation (TMS). Several single and double-pulse protocols allow an assessment of the excitability as well as its inhibitory or faciliatory modulation (17–21).

To date, only a few studies have investigated the effect of taVNS on the motor system using TMS so far. Capone et al. (22) demonstrated an increase in inhibition, assessed with the short intracortical inhibition (SICI) in a small group of 10 participants. Additionally, a study by Mertens et al. (23) revealed an altered resting motor threshold (RMT) in a small group of 8 subjects. In a very recent study, the increasing SICI after taVNS could be confirmed in 30 healthy subjects using Bayesian statistics, but differences in taVNS intensity and data analysis hamper the generalizability of the results (24).

Hence, current evidence that taVNS might modulate the excitability of the motor cortex can be considered rather limited. Thus, the aim of this study is to provide an in-depth examination of the modulatory effect of taVNS on cerebral motor cortex excitability in healthy study subjects.

This protocol is intended to describe the appropriate background, methods, and procedures for the proposed study. A summary of study details as well as differences between this study protocol and prior studies can be seen in Table 1.

**Table 1:** Study details and previous studies.

		**Planned study**
Capone et al. (22)	−10 healthy participants - Randomized placebo-controlled double-blind study - 60 min taVNS - Motor threshold, motor evoked potential amplitude, recruitment curve, SICI	−30 healthy participants - Randomized, sham-controlled, double blind study - 30 min taVNS - RMT, AMT, SICI, ICF - Tolerance and perception threshold verum and sham taVNS - TMS-EMG coupled
Mertens et al. (23)	−15 healthy male participants - TMS-EMG and TMS-EEG - Motor evoked potentals, TMS-evoked potentials, single and paired pulse TMS - 60 min taVNS - Maximun tolerated stimulation	
Van Midden et al. (24)	−30 healthy participants - Double-blind, sham controlled - taVNS at 100 Hz - SICI, SAI	

## Methods and analysis

### Study design

This is a single-center, prospective, double blind, sham-controlled trial evaluating the effect of taVNS on motor cortex excitability. To this end, this study uses four stimulation conditions: taVNS/sham stimulation at perception threshold and taVNS/sham stimulation at tolerance threshold applied in randomized order using a within subject design.

### Study population and recruitment

In total, 30 subjects will be included in the study. The subjects for the planned study are recruited around the University of Greifswald. The subjects will be recruited for the study by means of a verbal announcement. Due to the time required for the study, all participants will receive an expense allowance of 100 euros after completion of the study for their time.

### Inclusion/exclusion criteria

Participants will be eligible for the study according to the following criteria (see Table 2):

**Table 2:** Inclusion and exclusion criteria.

**Inclusion criteria**	**Exclusion criteria**
Age > 18 years and < 60 years	History of neurodegenerative or other neurological disorders; epilepsy or history of seizures.
	Persistent cardiovascular diseases or skin diseases
	Intake of long-term medication of any kind (except for oral contraception)
	Implanted medical devices or metal
	Pregnancy

Eligible participants will provide written informed consent prior study inclusion and randomization.

### Interventions

The study consists of a structural magnetic resonance imaging (MRI) and of four experimental sessions in total, during which either verum or sham taVNS will be administered at different stimulation intensities. TMS motor physiological measurements will be conducted before and after each taVNS session.

TMS and taVNS will be performed by two administrators (TH or PK). The TMS administrator will be blinded to the taVNS intervention condition (verum or sham), and vice versa. Data collection will take place in the TMS lab. Each measurement (TMS/taVNS) will be performed by only one investigator (TH/PK), with the other investigator being in the room next to the lab to avoid unblinding. The participant will also be instructed to not tell anything about the taVNS to the investigator performing the TMS.

In sum, the administrator applying taVNS will have no knowledge about the TMS parameters. There will be a minimum interval of 1 week between each experimental visit to reduce potential carry-over effects of the previous taVNS.

#### Structural MRI

High-resolution structural MRI (3 Tesla Siemens Magnetom Verio, 32-channel head coil, T1-weighted sequence with 176 sagittal slices, voxel size 1 × 1 × 1 mm^3^) will be acquired and will be 3D-reconstructed to be used for neuronavigation during the TMS procedures. This allows for accurate replication of TMS stimulation target between experimental visits using MR-navigated TMS.

#### TMS administration

First, neuronavigation will be performed with a stereo-tactical infrared optical-tracking Polaris camera (Polaris System, Northern Digital, Waterloo, Ontario, Canada) and BrainSight (BrainSight TMS, Rogue Research Inc.). Neuronavigated TMS will be applied via two Magstim 200 stimulators connected to form a BiStim unit (Magstim Company, Dyfed, UK) before and after taVNS application. Here, a figure-of-eight coil (70 mm in wing diameter) will be held tangentially over the scalp with orientation of the handle angled 45 degrees in the sagittal plane. The coil will be positioned that a maximum-evoked motor evoked potential (MEP) can be derived (motor hotspot). The cortical area stimulated by the TMS coil will be digitally marked on the MRI and stored in the neuronavigation program to guarantee precise, navigation-based stimulation on all examination days. Baseline TMS will take place on each experimental visit day, followed by verum or sham taVNS and will be repeated immediately after taVNS administration.

Surface electromyography (EMG) will be derived over the right and left interosseus dorsalis I muscles using Ag/AgCl electrodes (Ambu, Ballerup, Denmark) measuring 10 mm in diameter. The EMG signal will be amplified (CED 1902; Cambridge Electronic Design, Cambridge, United Kingdom), band-pass filtered (20–1,000 Hz), recorded with a temporal resolution of 2 kHz (CED 1401), and stored for off-line analyses (Signal V4.09). During the TMS examination, the patient will sit relaxed in an examination chair while the hand will be positioned on a pillow for measuring. Measurements from the EMG will be used to assess motor physiology.

The following motor physiology outcome measures will be collected: resting motor threshold (RMT), active motor threshold (AMT), input-output curve [recruitment curve; (RC)], short intracortical inhibition (SICI), intracortical facilitation (ICF).

The RMT is the minimum applied stimulation intensity to derive MEPs with at least a 50 μV amplitude level in four out of eight trials in relaxation.

The AMT is the minimum-applied stimulation strength to derive visible MEPs in four out of eight trials with minimal tension on the target muscle (abduction of the index finger) with ~100 μV EMG.

For evaluation of the RC, 10 × stimulation each at an intensity of 90, 100, 110, 120, 130, 140, 150, and 160% RMT will be presented in an pseudorandomized order.

For the double pulse protocols, the intensity of the test stimulus is chosen to be at the maximum slope of the RC. The conditioning stimulus (CS) for SICI and ICF is set to 80% RMT. Here, the interstimulation interval (ISI) between the conditioning stimuli and the following test stimulus is 2 ms for inhibition, and, respectively, 10 ms for facilitation. In total, 15 single pulses and 30 paired pulses (15 each of SICI and ICF) will be administered in a pseudorandomized manner and amplitude magnitudes will be recorded and stored for off-line analysis after each trial.

The analysis will be conducted using Signal software Version 4.11 (Cambridge Electronic Design, Cambridge, UK). Fitting of RC will be based on the 10 trials per stimulus intensity using a sigmoid Boltzman function to each individual RC, thereby estimating MEPmax (plateau of RC), SLOPEmax (slope of RC, straight line fitted to RC at its inflection point) and stimulus intensity s50 to obtain a response of 50% of the maximum (25).

SICI is expressed as a percentage as % SICI = 100 – (C/NC × 100), where C and NC represent the corresponding mean of the conditioned (C) or non-conditioned (NC) test stimulus MEPs. ICF is expressed as % ICF = (C/NC × 100) – 100. Parameters for both hemispheres are determined.

#### taVNS administration

Verum or sham taVNS will be performed using a tVNS^®^L-device (tVNS Technologies GmbH, Erlangen, Germany). The stimulation stimulus will be applied via two round, iridium covered, 2 mm diameter titanium electrodes, which will be attached to the subject's left ear in different positions (taVNS: cymba conchae; sham: earlobe; before taVNS will be attached, the skin will be prepared using an alcohol wipe to clean the skin and remove skin particles). Left sided taVNS will be applied to avoid the theoretically possible effect on the heart rate by stimulating vagal parasympathetic fibers (25). During the study, each subject undergoes a total of four different taVNS examination modalities (verum or sham taVNS at varying intensities; each patient will be assessed the same time of day to prevent circadian bias). On the first day of the study, the individual perception threshold and tolerance threshold of taVNS will be determined for each subject after the first TMS. During this procedure, the subject will sit on a chair while the taVNS device is attached to the left ear (stimulation site: cymba conchae). Using the tVNS Research App (tVNS Technologies GmbH, Erlangen, Germany), an increasing current will be applied (100 μA steps each; minimum: 100 μA, maximum: 5,000 μA). The patient will indicate the point at which he or she feels stimulation for the first time. This corresponds to the perception threshold of the taVNS. The perception threshold will be noted and stored for further study sessions. After the determination of the perception threshold, the current will be increased by 100 μA steps until the subject perceives the stimulation as painful. The intensity will then be reduced again until the stimulation is well-tolerated by the subject (defined as a strong tingling sensation, without pain perception). This procedure will be repeated a second time. Subsequently, the main value of the four values (pain–strong tingling—pain—strong tingling) will be calculated. The result corresponds to the tolerance threshold. In a randomized sequence, one of the two stimulation intensities will be applied either to the cymba conchae (verum stimulation) or to the earlobe (sham stimulation). In total, this results in four different types of application: verum taVNS at the perception threshold, verum taVNS at the tolerance threshold, sham taVNS at the perception threshold, and sham taVNS at the tolerance threshold. In every session, one out of the four application types will be chosen in a randomized order.

Stimulation, regardless of intensity, will be delivered continuously for 30 min (pulse width: 250 μs, stimulation frequency: 25 Hz, 30 s on/30 s off) to the left ear of participants. Motion threshold: off, ramp-up: off, Buzzer: off.

The corresponding settings will be transferred to the device via the tVNS Research App (tVNS Technologies GmbH, Erlangen, Germany) and applied accordingly. For this purpose, a bluetooth connection with a mobile device will be established to the device. The data will be entered via the smartphone while the device will be stimulated via a type of headphones.

### Randomization

A lottery procedure will be used to randomize the order of the taVNS. The four possible intervention methods (verum/sham taVNS x perception and pain threshold) will be written down and placed in an envelope, so it is not visible from the outside what is written on each slip of paper. The examiner performing the taVNS will use the measure that is written on the paper. One measure per examination day will be administered, so each subject completes each of the four measures.

### Study schedule

A summary of the examination procedure for the first, as well as for the following three examination days is shown in Table 3.

**Table 3:** Study schedule.

**Session 1**	**Session 2-4**
**Structural MRI (T1)**	
Navigated TMS	Navigated TMS
RMT/AMT	RMT/AMT
RC	RC
SICI/ICF	SICI/ICF
**Determination of tVNS perception threshold and**	
**discomfort threshold**	
One of the interventions, randomized	One of the interventions, randomized
Verum taVNS (perception threshold)	Verum taVNS (perception threshold)
Verum taVNS (tolerance threshold)	Verum taVNS (tolerance threshold)
Sham taVNS (perception threshold)	Sham taVNS (perception threshold)
Sham taVNS (tolerance threshold)	Sham taVNS (tolerance threshold)
Navigated TMS	Navigated TMS
RMT/AMT	RMT/AMT
RC	RC
SICI/ICF	SICI/ICF

### Outcomes

#### Primary outcome

The primary outcome measurement of cortical excitability will be the post-pre change of SICI in relation to the stimulation method (sham vs. verum).

#### Exploratory outcomes

Exploratory outcome measurements will be the post-pre changes of the TMS parameters (ICF, RMT, AMT, RC), the effect of taVNS intensity on the TMS parameters as well as side effect differences between verum and sham stimulation.

### Statistical analysis

#### Sample size

Overall, only a few studies investigating cerebral motor excitability after taVNS stimulation exist (22–24). Therefore, the determination of the effect size regarding the effect of taVNS on cortical excitability can only be determined inadequately. The total sample size of 30 subjects was determined based on the sample sizes of the mentioned studies (Capone 10 participants, Mertens 15 participants, van Midden 30 participants) (22–24). According to our a priori power analysis using G-Power, this sample size is sufficient to detect small to medium effect sizes (*f* = 0.18) with a power of 0.80.

#### Data analysis

According to our primary outcome measurement, a three-way repeated measure ANOVA with factors time (pre, post), stimulus (verum, sham) as well as stimulus intensity (tolerance, perception threshold) will be performed.

Additional ANOVAs for each exploratory outcome variable will be calculated using the same ANOVA model.

In case of violation of requirements for parametric methods, data will be transformed before analysis or appropriate non-parametric tests will be conducted. Data analysis will be conducted using IBM SPSS Statistics for Windows Version 25 (IBM), MATLAB (MathWorks, 2016) and R software (http://www.R-project.org).

## Discussion

To date, there has been a gap in understanding whether taVNS modulates cortical excitability and its underlying mechanism. For the first time, this prospective trial will investigate the effects of taVNS on motor cortex excitability as well as on distinct GABAergic and glutamergic pathways in human motor cortex in a double blinded, SHAM controlled, study design.

Non-invasive taVNS is a widely used method in biopsychological research with increasing evidence of effectiveness also in neurological diseases [for a review see for example Yap et al. (3) and (31)]. Interestingly, several studies focused on motor outcome parameters, for example in stroke research (26–30), but only a very few studies have been conducted to investigate the influence of taVNS on cortical excitability itself (22–24).

There is a high methodological heterogeneity between these previous studies regarding the taVNS protocol, different number of participants, as well as different methods for data analysis, which significantly impairs the generalizability of the results.

We will conduct this study to broaden the knowledge about the modulatory effect of taVNS. We will also reach a high data quality using a prospective, double blind, randomized, sham-controlled within subject trial with taVNS at different intensities. Our results will help to understand the physiological role of vagus nerve stimulation on the motor system itself, which makes it possible to better understand the application of this method in neurological conditions like stroke.

## Ethics statement

The studies involving humans were approved by Ethics Committee of the University Medical Center Greifswald (study reference number: BB048/22). The studies were conducted in accordance with the local legislation and institutional requirements. The participants provided their written informed consent to participate in this study.

## Author contributions

TH: Conceptualization, Investigation, Methodology, Writing—original draft, Writing—review & editing. PK: Conceptualization, Methodology, Investigation, Writing—original draft. SS: Writing—original draft. CS: Writing—original draft. NK: Writing—original draft. BB: Conceptualization, Writing—original draft. MW: Conceptualization, Project administration, Writing—review & editing. MG: Conceptualization, Investigation, Methodology, Project administration, Supervision, Writing—review & editing.
